# JCDB: a comprehensive knowledge base for *Jatropha curcas*, an emerging model for woody energy plants

**DOI:** 10.1186/s12864-019-6356-z

**Published:** 2019-12-24

**Authors:** Xuan Zhang, Bang-Zhen Pan, Maosheng Chen, Wen Chen, Jing Li, Zeng-Fu Xu, Changning Liu

**Affiliations:** 1 CAS Key Laboratory of Tropical Plant Resources and Sustainable Use, Xishuangbanna Tropical Botanical Garden, The Innovative Academy of Seed Design, Chinese Academy of Sciences, Menglun, Mengla Yunnan, 666303 China; 2 College of Life Sciences, University of Chinese Academy of Sciences, Beijing, 100049 China; 3 Center of Economic Botany, Core Botanical Gardens, Chinese Academy of Sciences, Menglun, Mengla Yunnan, 666303 China

**Keywords:** *Jatropha curcas*, Woody energy plant, Functional genomics, Database

## Abstract

**Background:**

*Jatropha curcas* is an oil-bearing plant, and has seeds with high oil content (~ 40%). Several advantages, such as easy genetic transformation and short generation duration, have led to the emergence of *J. curcas* as a model for woody energy plants. With the development of high-throughput sequencing, the genome of *Jatropha curcas* has been sequenced by different groups and a mass of transcriptome data was released. How to integrate and analyze these omics data is crucial for functional genomics research on *J. curcas*.

**Results:**

By establishing pipelines for processing novel gene identification, gene function annotation, and gene network construction, we systematically integrated and analyzed a series of *J. curcas* transcriptome data. Based on these data, we constructed a *J. curcas* database (JCDB), which not only includes general gene information, gene functional annotation, gene interaction networks, and gene expression matrices but also provides tools for browsing, searching, and downloading data, as well as online BLAST, the JBrowse genome browser, ID conversion, heatmaps, and gene network analysis tools.

**Conclusions:**

JCDB is the most comprehensive and well annotated knowledge base for *J. curcas*. We believe it will make a valuable contribution to the functional genomics study of *J. curcas*. The database is accessible at http://jcdb.xtbg.ac.cn.

## Background

*Jatropha curcas* is a perennial shrub belonging to the Euphorbiaceae family. It is a tropical species that is native to Mexico and Central America and now thrives in Latin America, Africa, India, and South East Asia [1–5]. As a multi-functional plant, it has been used in traditional medicine and for hedges, animal feed, and firewood [6–9]. With the gradual depletion and cost escalation of fossil energy resources, *J. curcas* is now attracting much attention for its potential use for biofuel production, because of its high seed oil content (the seeds of *J. curcas* contain ~ 40% oil) [10], easy propagation, rapid growth, and ability to grow in a wide range of conditions, including degraded, sodic, alkaline, and contaminated soils [7, 11].

*J. curcas* has a relatively small genome, which is organized in 22 chromosomes (2n) [12]. The *J. curcas* genome has been sequenced by four groups worldwide [13–17]. For the RefSeq representative version from the Wu laboratory, the assembled genome is 320.5 Mb [15]. *J. curcas* also has several advantages, including easy genetic transformation and short generation duration, which make it an attractive wood energy model plant for function genome analysis, particular among the Euphorbiaceae [18–20]. *J. curcas* is also a potential model for studies of flower sex determination in monoecious trees, as most *J. curcas* germplasms are monoecious, bearing male and female flowers on the same inflorescence [21, 22].

In recent years, there have been significant advances in the application of transcriptome analysis to *J. curcas* [22–31]. Using bioinformatics tools and a comprehensive knowledge database to integrate all these genome and transcriptome data is crucial for further functional genomics research on *J. curcas.* Advances in *J. curcas* research have led to the creation of several *J. curcas* genetic information resources. For instance, the Jatropha Genome Database (JAT_r4.5) focuses on the *J. curcas* genome sequence and annotation [13], and KaPPA-View4 is a KEGG (Kyoto Encyclopedia of Genes and Genomes) pathway viewer for *J. curcas* [32]. Although each of these resources provides valuable information, there is a lack of database unification and integration of the *J. curcas* genome and transcriptome with a broad set of multi-omics analysis results, such as gene functional annotation, gene expression matrices, and gene interaction networks.

In this study, we constructed a *J. curcas* database (JCDB) that is dedicated to providing a comprehensive platform for *J. curcas* functional genomics research. By establishing pipelines for processing novel gene identification, gene function annotation, gene expression level quantification, and gene network construction, we systematically integrated and analyzed a series of *J. curcas* transcriptome data, which were used to generate JCDB. The database includes general gene information (including genomic coordinates and sequences), gene functional annotation (including gene ontology (GO), KEGG, Pfam, and InterPro), gene interaction networks (gene co-expression and protein-protein interaction (PPI) networks), and gene expression matrices. We also provide tools for browsing, searching, and downloading all data, as well as user-friendly web services such as BLAST, the JBrowse genome browser, ID conversion, heatmaps, and gene network analysis tools. In the case studies presented here, we demonstrate the possibility of using JCDB to mine genes related to flowering and lipid synthesis pathways in *J. curcas*. We believe that JCDB represents a valuable and unique resource for further functional genomics studies of *J. curcas*.

## Construction and content

### Transcriptome data retrieving and processing

**Fig. 1:**
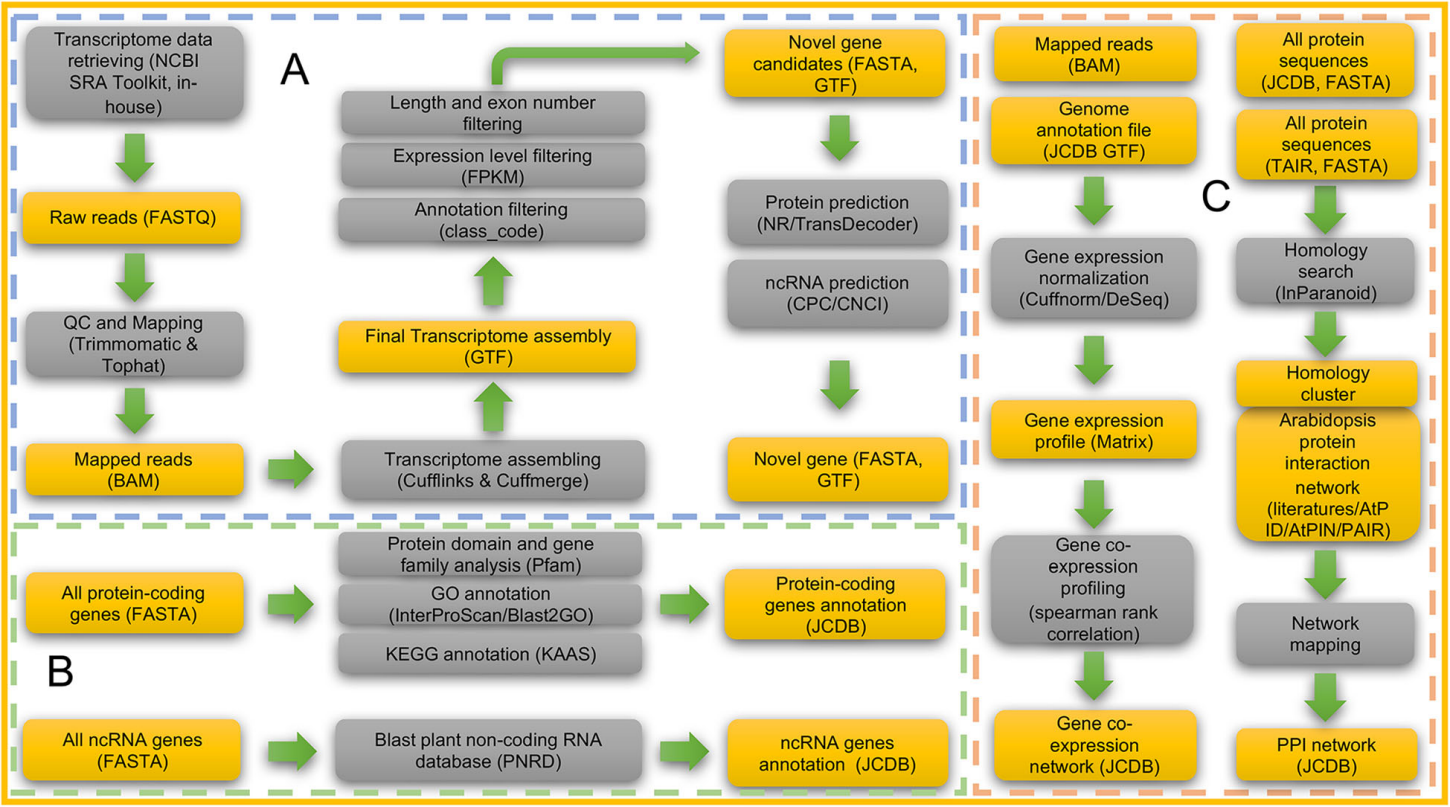
JCDB pipelines for data retrieval and processing. **a** Novel gene discovery pipeline. **b** Coding and non-coding gene (ncRNA) annotation pipeline. **c** Gene co-expression and PPI network construction pipeline

To acquire comprehensive genomic information for *J. curcas*, we developed a pipeline for transcriptome data collection, integration, and novel gene identification, including non-coding RNAs (Fig. 1a). First, publicly available transcriptome data of *J. curcas* were downloaded from NCBI’s Sequence Read Archive (SRA) database. Detailed information was collated for each sample, including experimental description, organizational information, and references. (Additional file 1). The SRA data was dumped into the FASTQ format using the fastq-dump utility from the NCBI SRA Toolkit v.2.5.2 [33]. Raw reads were quality trimmed using Trimmomatic (version 0.32) with parameters “LEADING:20 TRAILING:20 MINLEN:36” [34]. Then, all clean reads were mapped onto the *J. curcas* genome (JatCur_1.0) [15] using TopHat 2 (version 2.1.0), with default parameters except maximum intron length, which was set to 20,000 bp [35]. Next, the mapped reads were assembled using Cufflinks (version 2.2.1) with the RefSeq genome as a guide, and a combined transcriptome assembly was generated using Cuffmerge [36]. Finally, genes that were identified by Cuffcompare as non-overlapping with known genes, having more than one exon, longer than 200 bp, and with FPKM (fragments per kilobase per million) greater than 0.1 were considered as novel gene candidates.

### Novel protein-coding and non-coding gene identification

As shown in Fig. 1a, novel transcript sequences were first used as query for a BLASTX search against the NCBI non-redundant protein (NR) database with default parameters. Then, open reading frames (ORFs) of these matches were identified using TransDecoder v4.1.0 (https://github.com/TransDecoder/TransDecoder). Matches with a completed ORF were annotated as protein-coding genes. Non-coding genes were further identified using CPC (Version 0.9-r2) [37] and CNCI (Version 2) [38] among the genes not matching the NCBI NR database. The remaining genes were annotated as transcripts of unknown coding potential (TUCPs).

### Protein-coding and novel non-coding gene annotation

All the protein-coding and novel non-coding genes in JCDB were annotated using the in-house gene annotation pipeline (Fig. 1b). For the annotation of protein-coding genes, Pfam [39] was used for protein domain and gene family analysis. GO annotations were assigned using InterProScan [40] and Blast2GO [41]. KEGG annotations were assigned using the online service KAAS [42]. For the annotation of novel non-coding genes, we downloaded all small non-coding RNA and long non-coding RNA (lncRNA) sequences from the plant ncRNA database PNRD [43] and annotated the JCDB novel non-coding genes using a BLAST search with default parameters. In total, there were 27 novel non-coding genes with BLAST hits to PNRD, including 22 microRNA (miRNA) host genes, two long intergenic non-coding RNAs (lincRNAs), and three lncRNAs of unknown type.

### Co-expression network construction

As shown in Fig. 1c, for conventional RNA-Seq data, gene expression profiles were identified and normalized using Cuffnorm [36]. For digital gene expression data, read count tables were created using htseq-count in the HTSeq toolkit [44] and then normalized using the DESeq method [45]. The two types of expression matrix were merged and normalized again using the upper-quartile method [44]. A gene co-expression network was constructed using the Spearman’s rank correlation coefficients of gene pairs across the samples. Gene pairs with correlation value higher than 0.6 and adjusted *P*-value less than 0.01 were regarded as showing co-expression.

### Protein-protein interaction network construction

Arabidopsis protein interactions were collected from the literature [46–48] and from three databases (AtPID 5.0 [49], AtPIN 9.0 [50], and PAIR 3.0 [51]), giving a total of 18,037 Arabidopsis genes and 241,468 interactions. Arabidopsis protein sequences were downloaded from TAIR10 [52]. The pairwise similarity matching tool InParanoid [53] with default settings was used to find orthologous groups between the *J. curcas* and Arabidopsis proteomes. The *J. curcas* PPI network was inferred from the Arabidopsis PPI network [46–51] by homology mapping (Fig. 1c).

### System implementation

The JCDB server was built using Apache/2.4.6 (CentOS), PHP (version 5.4.16), and relational database MySQL (version 5.5.48). The entity relationship diagram is provided in Additional file 2. The physical server was a 4 Intel(R) Xeon(R) CPU E5–2640 v3 @ 2.60 GHz with 8 GB RAM. All data and information were stored in MySQL tables to facilitate efficient management, search, and display. A combination of Thinkphp (version 3.2), Bootstrap (version 3.3.7), and JQuery (version 3.3.7) were used to construct the website. The network was visualized using Cytoscape.js (version 3.8).

## Utility and discussion

### Search JCDB

**Fig. 2:**
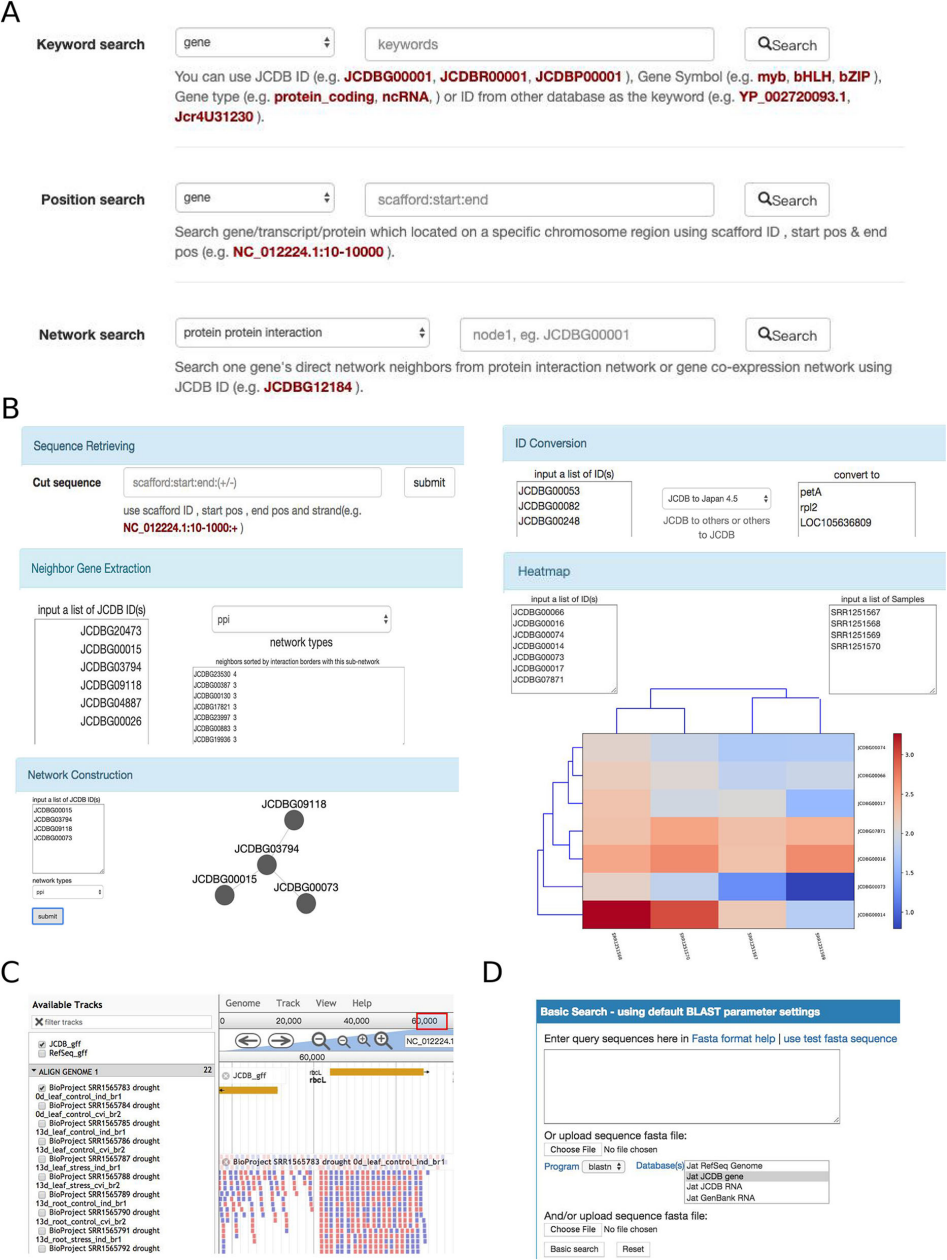
Screenshots of the JCDB online tools. **a** Keyword search, position search, and network search. **b** JCDBtools, the web-based toolkit. **c** JBrowse, the genome browser. **d** Online BLAST search

The ‘Search page of JCDB (Fig. 2a) provides three different types of search services. ‘Keyword Search’ uses keywords including gene types (such as protein_coding and ncRNA), gene symbols (such as bZIP, myb, and bHLH), and gene/transcript/protein IDs (such as JCDBG00001, JCDBR00001, and JCDBP00001) from JCDB or other databases (such as RefSeq, JAT_r4.5, and GenBank). ‘Position Search’ finds genes/transcripts/proteins located in one specific genomic region specified by the users. ‘Network Search’ provides a gene’s direct network neighbors in the PPI or co-expression network.

### JCDBTools

JCDBTools is a web-based toolkit that provides five tools to help molecular biologists use JCDB more efficiently (Fig. 2b). ‘Sequence Retrieving’ can be used to retrieve genome sequences by providing genomic coordinates. ‘ID Conversion’ converts gene/transcript/protein IDs between JCDB and other databases (including RefSeq, JAT_r4.5, and GenBank). ‘Heatmap’ can be used to retrieve the gene expression patterns of a group of genes from different samples. ‘Network Construction’ can be used to extract a sub-network for user-specified genes from the global PPI or co-expression network. ‘Neighbor Gene Extraction’ can be used to extract the nearest neighbors of a sub-network in the global PPI or co-expression network.

### JBrowse

JCDB integrates genome browser JBrowse [54] to provide easy-to-use panning and zooming navigation of the *J. curcas* reference genome (Fig. 2c). JBrowse includes various tracks, such as the *J. curcas* genome sequence, gene annotation GFF files from JCDB and RefSeq, and transcriptome-aligned BAM files for different samples.

### BLAST service

The BLAST server (Fig. 2d) was implemented using ViroBLAST [55], which is a user-friendly tool for interfacing with the command-line NCBI BLAST+ toolkits. For user convenience, JCDB BLAST provides nucleotide databases (RefSeq genome/RNA, JCDB gene/RNA, and GenBank RNA/CDS) and protein databases (JCDB Protein, GenBank Protein, and RefSeq Protein).

### Browse JCDB

**Fig. 3:**
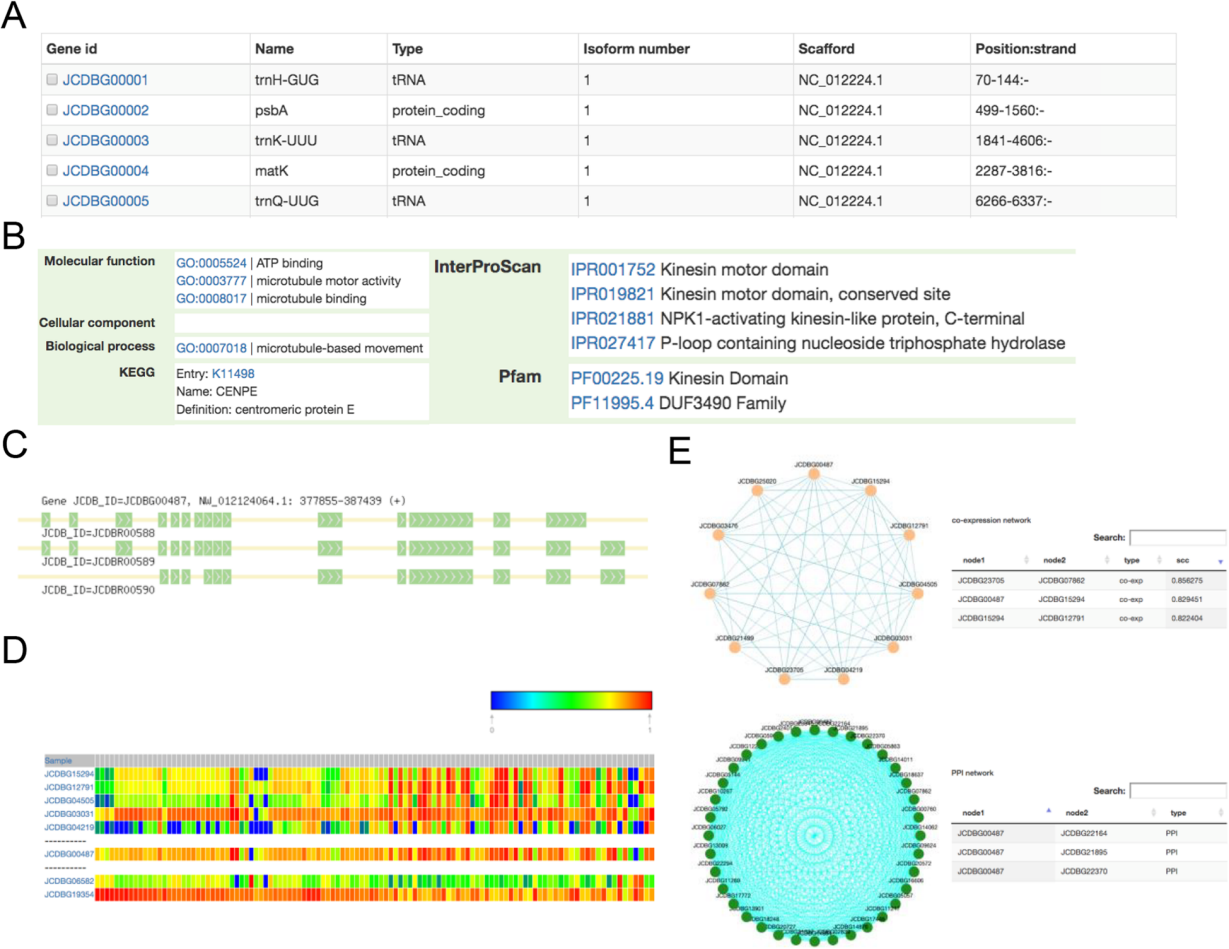
Screenshots of the browse and detail information pages. **a** The Browse page. **b** Detailed gene functional annotations. **c** Gene structural information. **d** Gene expression heatmap. **e** Gene co-expression network and PPI network

Users can browse all JCDB genes directly on the ‘Browse’ page (Fig. 3a), which provides basic annotations for each gene, such as gene name, gene type, and genomic location. Users can also select and download FASTA files for genes if required. Detailed information page for a specific gene can be accessed by clicking on the gene ID. For each gene, JCDB aims to provide as much comprehensive information as possible, including detailed GO, KEGG, InterPro, and Pfam functional annotations (Fig. 3b); structural information for each gene isoform (Fig. 3c); gene expression heatmaps (Fig. 3d); and co-expression and PPI sub-networks (Fig. 3e). In the gene expression heatmap panel, users can select the number of co-expressed genes that they want to display. In the gene sub-network panel, users can click and drag each gene node to move it, or click each gene ID to redirect to its detail page. The network is also displayed as a table on the right-hand side with a search function. Users can sort the table by column.

### Database statistics

Statistics for JCDB are summarized in Table 1. The current database release contains a total of 25,297 genes and 33,785 transcripts, including protein-coding genes (22,446, about 89%), non-coding genes (2391, about 9%), and TUCP genes (460, about 2%). Compared with existing *J. curcas* databases [13, 15, 32], JCDB includes more non-coding genes and more annotation information, as well as unique gene networks and expression profiles (Table 2). In JCDB, about 58, 40, and 74% of genes have GO, KEGG, and Pfam annotations, respectively; there are also about 90% genes in the co-expression network, 38% genes in the PPI network, and 114 expression profiles for 25,297 genes. Users can freely download all the above annotation files via the Download page.

**Table 1:** Gene statistics and data integrated in JCDB

Category	Number
Genes/transcripts	
All	25,297/33,785
Protein-coding	22,446 (89%)
Non-coding	2391 (9%)
TUCP	460 (2%)
Gene annotation	
Gene ontology	14,714 (58%)
KEGG pathway	10,217 (40%)
Pfam domain	18,829 (74%)
Genes in network	
Co-expression network	22,749 (90%)
PPI network	9602 (38%)
Expression profiles	114

**Table 2:** Comparison of gene annotations in JCDB with other Jatropha databases

Database	Protein	ncRNA	GO	KEGG	Pfam	Network	Expression
JAT_r4.5 [13]	30,203	0	x	x	x	x	x
KaPPA-View4 -Jatropha [32]	40,929	0	x	√	x	√	√
RefSeq [15]	21,574	2013	x	x	x	x	x
JCDB	22,446	2391	√	√	√	√	√

### Case studies

JCDB provides a comprehensive platform for *J. curcas* functional genomics research by integrating information from various sources, including gene functional annotations and gene interaction networks, and various tools including BLAST search and gene network analysis. Here, we demonstrate the use of the information and tools provided by JCDB to mine some important gene pathways in *J. curcas*.

**Fig. 4:**
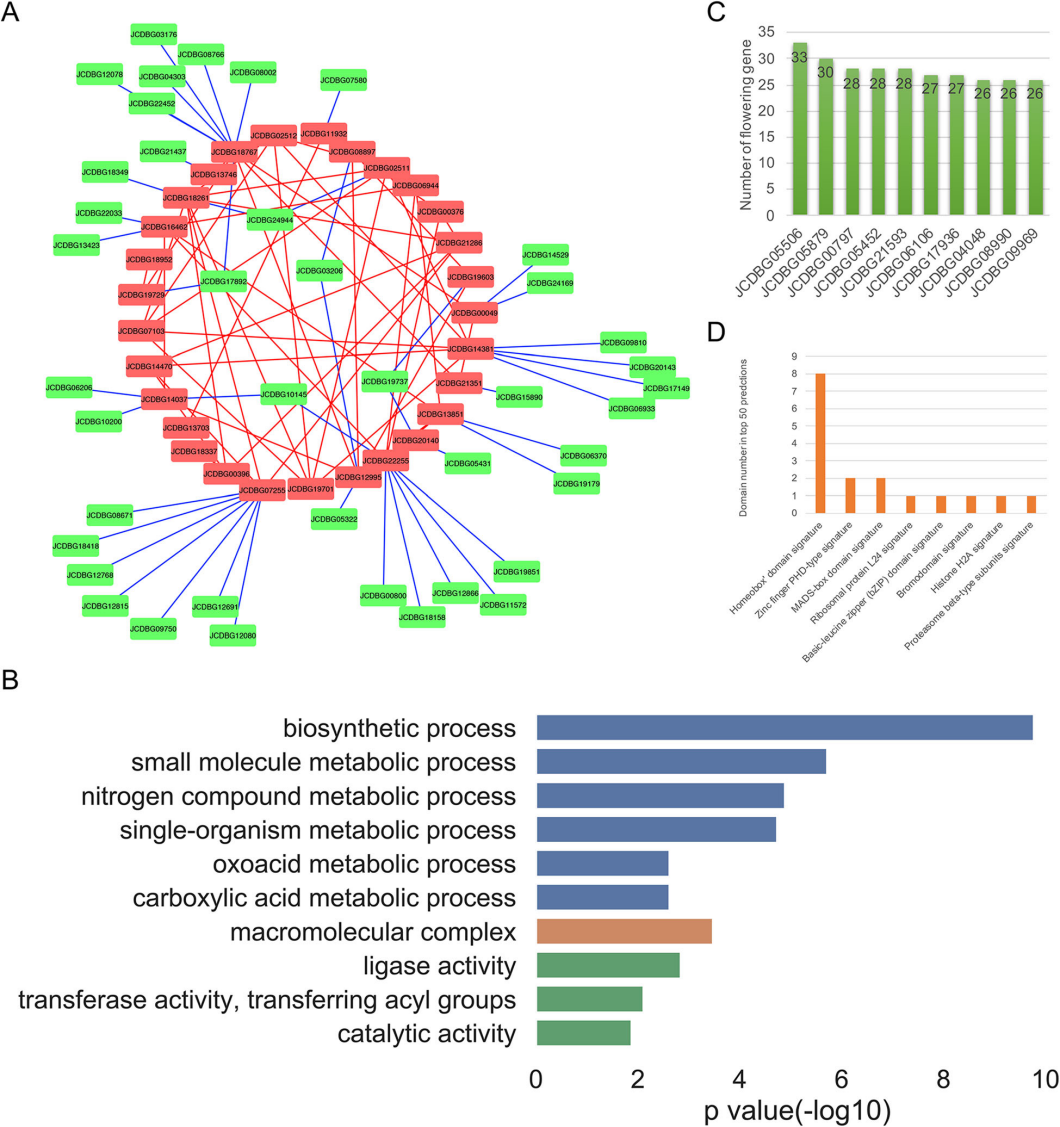
Case studies: gene function prediction using JCDBTools. **a** Sub-network of oil-related genes in *J. curcas* (red: known, green: prediction). **b** GO enrichment analysis of predicted oil-related genes (blue: BP, orange: CC, green: MF). **c** Numbers of known flowering-related genes interacting with predicted flowering-related genes (top 10). **d** Protein domain information for the top 50 predicted flowering-related genes

In order to better understand the genetic control of fatty acid and lipid biosynthesis in *J. curcas*, we collected 132 oil-related genes from *Arabidopsis* and identified oil-related gene candidates in *J. curcas* using the JCDB BLAST search. Using the ‘Network Construction’ function in JCDBTools, we obtained a *J. curcas* oil-related gene sub-network, which showed that these *J. curcas* oil-related genes were closely connected (Fig. 4a). We also used the ‘Neighbor Gene Extraction’ function in JCDBTools to find *J. curcas*-specific oil-related genes. We first extracted all the nearest neighbors of the known oil-related genes and then retained those that interacted with known oil-related genes in both the PPI and co-expression networks. We examined the GO annotations of these *J. curcas* specific oil-related gene candidates using GOATOOLS [56] (Fig. 4b). Consistent with our assumption, these genes appeared to be related to oil synthesis. The top enriched GO terms for biological process (BP) included biosynthetic process, small molecule metabolic process, and oxoacid and carboxylic acid metabolic process; the top cellular component (CC) term was macromolecular complex; and the top molecular function (MF) terms were ligase activity, transferase activity, transferring acyl groups, and catalytic activity.

We also investigated the flowering-related pathway in *J. curcas*. By manually reviewing the published literature, we identified 303 flowering-related genes of *Arabidopsis*. Then, using the same method, a total of 187 flowering-related genes in *J. curcas* were identified through homologous search, and the nearest neighbors and sub-network of these known flowering-related genes were also obtained. In the sub-network, the *J. curcas*-specific flowering-related gene candidates were closely connected with the known flowering-related genes. All the top 10 candidates had more than 25 interactions, including JCDBG05506 (Fig. 4c). Searching for this gene in JCDB revealed that JCDBG05506 is a MADS-box protein, with annotations including “FLOWERING LOCUS C” and “transcription factor”. Furthermore, we counted the protein domain annotations of the top 50 *J. curcas*-specific flowering-related gene candidates and found eight genes containing a homeobox domain, as well as two genes containing the zinc finger PHD-type domain and two genes containing the MADS-box domain (Fig. 4d). All of these protein domains are reported to be related to flowering [56–58].

## Conclusions

The plant *J. curcas* has attracted much attention worldwide owing to its potential for biofuel production. However, current databases for *J. curcas* did not effectively integrate multiple data sources and lacked useful tools for data presentation and analysis, and thus could not meet the needs of functional genomics study. For these reasons, we built JCDB, an integrated knowledge base, which includes not only basic gene information but also gene functional annotations, gene expression profiles, and gene network information. JCDB also provides a user-friendly platform for data presentation and analysis, offering a variety of tools including BLAST, the JBrowse genome browser, and JCDBTools. JCDB is the most comprehensive and well-annotated database available for *J. curcas* functional genomics research. Future work will include developing new tools to assist users with in-depth exploration of JCDB. We believe JCDB will continue to provide a valuable and unique resource for *J. curcas* functional genomics studies.

## Supplementary information

**Additional file 1.** The transcriptome data sources of *Jatropha curcas*.

**Additional file 2.** The entity relationship diagram of JCDB.

## Data Availability

All data generated or analyzed during this study are included in this published article.

## Abbreviations

BP: Biological process
CC: Cellular component
GO: Gene ontology
JCDB: *Jatropha curcas* database
KEGG: Kyoto Encyclopedia of Genes and Genomes
lncRNA: Long non-coding RNA
MF: Molecular function
ORFs: Open reading frames
PPI: Protein-protein interaction
SRA: Sequence Read Archive
TUCPs: Transcripts of unknown coding potential
