# Supplementary figures and images for: JCDB: a comprehensive knowledge base for Jatropha curcas, an emerging model for woody energy plants
Source: BMC Genomics. 2019 Dec 24;20(Suppl 9):958. doi: 10.1186/s12864-019-6356-z (PMC6929279; doi:10.1186/s12864-019-6356-z)

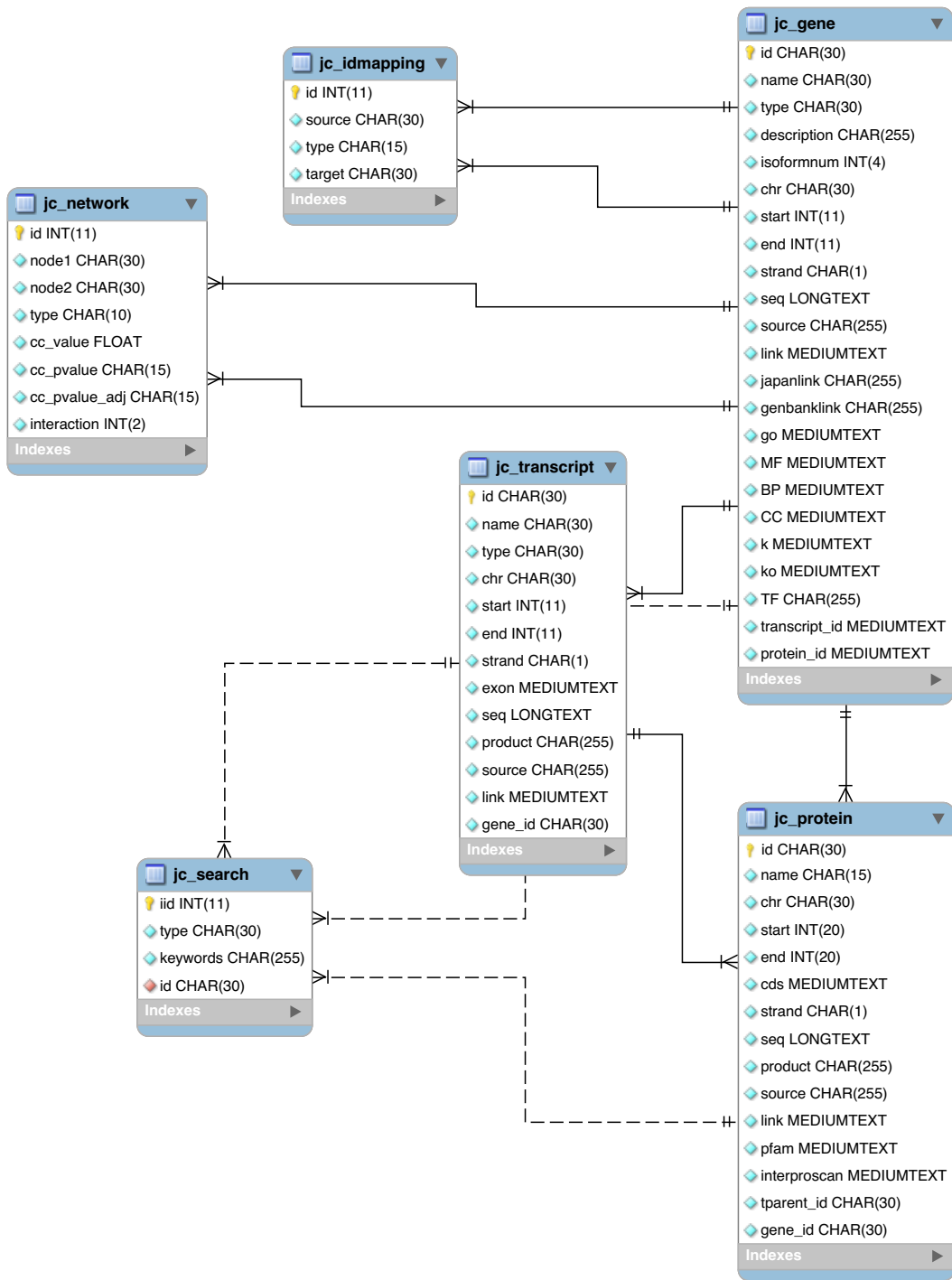

Supplement: Supplementary file 2 — Additional file 2. The entity relationship diagram of JCDB. [file 12864_2019_6356_MOESM2_ESM.pdf]
